# Therapeutic Drug Monitoring of Amikacin in Neutropenic Oncology Patients

**DOI:** 10.3390/antibiotics12020373

**Published:** 2023-02-11

**Authors:** Maria Aquino, Maria Tinoco, Joana Bicker, Amílcar Falcão, Marília Rocha, Ana Fortuna

**Affiliations:** 1 Laboratory of Pharmacology, Faculty of Pharmacy, University of Coimbra, 3000-548 Coimbra, Portugal; 2 CIBIT—Coimbra Institute for Biomedical Imaging and Translational Research, University of Coimbra, 3000-548 Coimbra, Portugal; 3 Centro Hospitalar e Universitário de Coimbra (CHUC, EPE), 3000-548 Coimbra, Portugal

**Keywords:** antibiotics, therapeutic drug monitoring, amikacin, tumor, neutropenia, pharmacokinetics

## Abstract

Amikacin is the antibiotic of choice for the treatment of Gram-negative infections, namely, those in neutropenic oncology patients. No populational pharmacokinetic studies are currently available reporting amikacin pharmacokinetics in neutropenic oncology patients despite their specific pathophysiological features and treatments. A large-scale retrospective study was herein conducted to specifically investigate the effects that tumor diseases have on the pharmacokinetic parameters of amikacin and identify whether chemotherapy, the lag time between administration of chemotherapy and amikacin, age and renal function contribute to amikacin pharmacokinetics in neutropenic cancer patients. A total of 1180 pharmacokinetic analysis from 629 neutropenic patients were enrolled. The daily dose administered to oncology patients was higher than that administered to non-oncology patients (*p* < 0.0001). No statistical differences were found in amikacin concentrations, probably because drug clearance was increased in cancer patients (*p* < 0.0001). Chemotherapy influenced amikacin pharmacokinetics and drug clearance decreased as the lag time enhanced. The elderly group revealed no statistical differences between the doses administered to both the oncology groups, suggesting that the impact of ageing is stronger than chemotherapy. Our research suggests that cancer patients require higher initial doses of amikacin, as well as when chemotherapy is received less than 30 days before amikacin treatment has started.

## 1. Introduction

Malignant tumor prevalence is drastically increasing worldwide, and it is expected to enhance even more with the growing and aging population [1,2]. Important unprecedented advances have been made in earlier cancer detection and pharmacological treatments that include, but are not limited to, chemotherapy and immunosuppressants, improving the quality of life and expanding the lifespan of oncologic patients. In fact, 70% of cancer survivors will be alive 5 or more years from diagnosis, and nearly 18% will survive 20 years or longer [3].

Notwithstanding, pharmacological treatments are associated with high toxicity as a result of their poor selectivity and strong side effects that have a negative impact on functional health. In particular, oncology patients, including pediatric patients, frequently experience episodes of prolonged neutropenia, which puts them at high risk of infection with significant morbidity and mortality [4,5,6]. The intensification of anti-tumor regimens enhances both the depth and length of neutropenia and endorses severe specific immune dysfunctions. Consequently, patient neutropenic state, together with pathological tumor evolution, involve a synergistic effect that increases the risk of developing bacterial infections, hospitalization, morbidity, mortality and treatment costs [7,8,9]. Clinical signs of neutropenia in oncological patients include a single axillary/oral temperature higher than 101.3 °F (38.5 °C) or a sustained temperature of 100.4 °F (38 °C) or higher for one hour, an absolute neutrophil count (ANC) lower than 500 cells/mm^3^ or an expected ANC decrease to less than 500 cells/mm^3^ within 48 h. Under these conditions, oncology patients must receive blood cultures and inpatient treatment with empiric antibiotics until they are afebrile for 48–72 h and ANC are at least 500 cells per mm^3^ for 72 h [7]. It is noteworthy that an emergent use of antibiotics improves survival rates [10,11,12,13].

As a supportive therapy that does not directly treat cancer but addresses treatment-related side effects, intravenous amikacin is prescribed as a first line antibiotic to combat life-threatening Gram-negative infections in neutropenic oncology patients [14]. Although this aminoglycoside is extensively used, the accurate determination of its optimal dosage is hampered by a marked intra- and inter-individual variability. Specifically, in spite of its negligible binding to plasma proteins and metabolism, amikacin displays a large pharmacokinetic variability influenced by maturational and pathophysiological conditions [15,16,17,18]. As recently deeply revised in [17], some population pharmacokinetic studies and pharmacokinetic/pharmacodynamic (PK/PD) models have been developed for amikacin in special populations, such as pediatrics and critically ill patients, in an attempt to improve the design of optimal dosing regimens. However, to the best of our knowledge, no populational pharmacokinetic studies are currently available reporting amikacin pharmacokinetics in neutropenic oncology patients in spite of their pathophysiological features and concomitant pharmacotherapies that inevitably interfere with drug pharmacokinetic, therapeutic and toxicity profiles [19].

The increasing burden of cancer on the capacity of healthcare systems and the need to reduce the negative impact of the disease and its treatment on the quality of life of cancer patients require the development of cancer care strategies that are driven by personalized system approaches. While survival from cancer has improved in all age groups, the functional health of cancer survivors is a clear unmet need that has not been adequately addressed [1]. In this context, it is important to guarantee that antibiotic treatment is accurately prescribed to each specific oncologic patient. Identifying the differences between non-neutropenic and neutropenic oncologic patients is required to initialize and maintain an effective and safe therapy with amikacin and, hence, prevent the development of bacterial infection and resistance.

Therapeutic drug monitoring (TDM) of plasma or serum concentrations of antibiotics, namely, amikacin, is regarded the most practical means of assessing adequate antibiotic exposure to provide a safe and effective therapy for all patients through the delivery of personalized antibiotic dosing schemes [20,21,22]. Amikacin TDM involves the determination and interpretation of its concentrations in plasma (or serum) followed by the estimation of its pharmacokinetic parameters, namely, the volume of distribution and the total clearance, to define the optimal pharmacological scheme and to optimize the therapy when pathological conditions or pharmacological treatment change [23,24]. Amikacin exhibits a concentration-dependent post-antibiotic effect [25], a low volume of distribution (0.3–0.4 L/kg) and a clearance correlated with patient renal function [26]. It has a narrow therapeutic range, considerable inter- and intra-individual pharmacokinetic variability, and there is a well-described relationship between its systemic exposure and clinical response, making TDM advisable even though the etiology underlying its pharmacokinetic variability is still unknown [27]. The peak concentration (C_max_) and trough concentration (C_min_) of amikacin are ascribed as references for drug efficacy and toxicity, respectively, and they depend on therapeutic regimen type [28]. Conventionally, aminoglycosides were administered by giving lower doses divided into two or three doses per day. However, since high residual concentrations are associated with nephrotoxicity, the extended interval dosing method has revealed to be advantageous because it employs a dosing interval of 24, 36 or 48 h that maximizes antibacterial efficacy and limits toxicity [29]. Under this regimen, the intervals are extended long enough to allow amikacin to be fully eliminated. In critically ill patients under an extended interval administration regimen, the optimal peak was established between 50 and 64 μg/mL [30] and the potential toxicity threshold was defined as a C_min_ of 3 μg/mL at maximum [31,32].

The primary aim of this study was to characterize the dose regimen of amikacin and its pharmacokinetics in neutropenic oncology patients, comparing both profiles with non-oncology neutropenic patients. In addition, the impact of demographic and anthropometric characteristics, chemotherapy and renal function on amikacin concentrations and pharmacokinetics was also investigated. At the end, new recommendations for the loading dose of amikacin in neutropenic oncology population are herein proposed.

## 2. Results

### 2.1. Patients Characteristics

**Figure 1:**
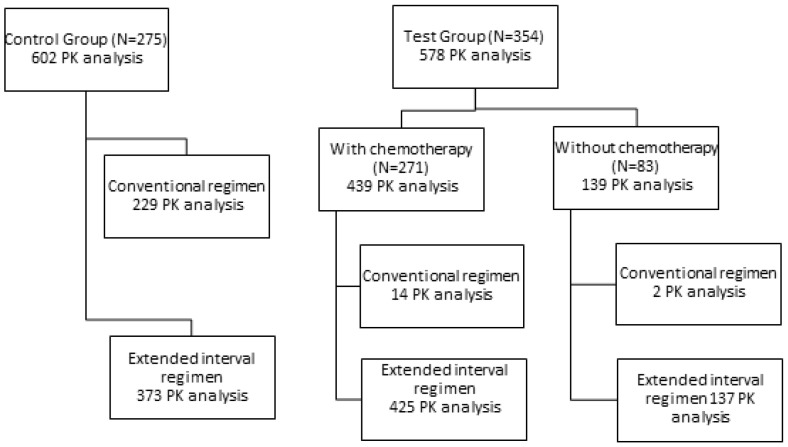
Pharmacokinetic (PK) analysis performed on each group herein investigated. Control Group includes neutropenic non-oncology patients, while Test Group includes neutropenic oncology patients that were distinguished in accordance with the last administered chemotherapy cycle: “with chemotherapy” includes those that received chemotherapy cycle; “without chemotherapy” includes patients that received other therapy but not chemotherapy. Each pharmacokinetic monitoring required the determination of the peak and trough concentrations and the estimation of the corresponding pharmacokinetic parameters.

A total of 1180 pharmacokinetic analysis from 629 neutropenic patients were evaluated and classified according to the study group displayed in Figure 1. Demographic, anthropometric and TDM characteristics of these patients are summarized in Table 1. With the exception of three patients (0.8%) from the test group that were admitted in the Surgery Service, all patients were admitted in Intern Medicine Service. The most prevalent oncologic disease found in the test group was acute myeloid leukemia (35.88%), followed by non-Hodgkin lymphoma (9.04%), multiple myeloma (6.50%), acute lymphoblastic leukemia (5.93%), and others with prevalence inferior to 5%. In general, 200 patients were diagnosed with leukemias (56.50%), 97 with lymphoma (27.40%) and 23 with multiple myeloma (6.50%).

Having analyzed the age mean values of both groups (Table 1), the difference between them is noteworthy (*p* < 0.0001). Age frequency distribution was very distinct, with the majority of the oncology patients included being between 45 and 64 years old, while the majority of non-oncology patients were between 65 and 85 years old. Therefore, and considering that amikacin is mainly eliminated in urine and that renal function is considerably affected in the elderly (≥65 years old), the influence of age and creatinine clearance on amikacin pharmacokinetics was herein investigated (Section 2.3.1 and Section 2.3.2).

Concerning anthropometric data, both populations presented similar mean values of body mass index (BMI, 24.31 and 25.58 kg/m^2^ for control and test group, respectively) and a similar frequency distribution (Table 1). Accordingly, most of the patients had BMI between 18.5 and 24.99 kg/m^2^, followed by the range of 25–29.99 kg/m^2^. Considering the World Health Organization (WHO), a BMI value between 18.5 and 25 kg/m^2^ is considered normal, while patients within the BMI range of 25–30 kg/m^2^ are overweight. Nevertheless, there were patients in all the BMI classes, emphasizing the inter-individual variability.

The mean daily dose of amikacin was 1170.80 mg (range, 500–2000 mg) in the test group and only 868.56 mg (range, 250–1750 mg) in the control group, highlighting the statistically significantly higher dose that is daily administered to oncology neutropenic patients comparatively to non-oncology neutropenic patients (*p* < 0.0001, Table 1). The wide dose ranges observed for both groups also evidence the high variability, which can be justified not only by inter-individual differences but also by the fact that two types of dose regimen have been herein enrolled (conventional and extended interval regimens). Indeed, C_max_ and C_min_ values depend on the posology regimen (Table 1), with extended interval administration exhibiting, as expected, lower values of C_min_ and higher values for C_max_.

Importantly, the extended interval dosing was associated with only 21.71% and 8.540% of oncology patients achieving target C_max_ values of 50 and 64 µg/mL, respectively. These percentages are slightly higher in non-oncology patients (23.59% and 11.26%, respectively). Regarding C_min_, 85.05% of the oncological patients revealed concentrations lower than 3 µg/mL, while only 63.00% of the non-oncology patients were within the pre-defined therapeutic range. These findings suggest that, despite receiving higher doses, neutropenic oncology patients remain at risk of developing subtherapeutic responses (given by the C_max_ values inferior to therapeutic range) with no safety concerns (given by the C_min_ within therapeutic range). Oncology patients exhibit higher amikacin clearance than the non-oncology population (3.709 vs. 2.778 mL/min, *p* < 0.0001, Table 1) and lower t_1/2_ (4.750 vs. 6.950 h, *p* < 0.0001). Both probably justify the reduced concentrations observed in oncology patients even though this group was administered with increased doses. The volume of saline injected per day was statistically superior in the oncology group (*p* < 0.0001).

### 2.2. Impact of Chemotherapy on Amikacin Pharmacokinetics

To evaluate the pharmacokinetics of amikacin in cancer patients, the population of the test group was divided considering whether the patients were or have been under chemotherapy treatments or not. The administered dose, plasma concentrations and pharmacokinetic parameters attained for both subpopulations and the control group were compared (Table 2).

The mean daily doses administered to the two oncologic subpopulations were very similar (*p* > 0.05) even though there was a tendency to administer higher doses to both oncology groups in relation to the control one (Table 2). Nevertheless, when under an extended interval regimen, patients without chemotherapy exhibited the highest amikacin C_max_ (*p* < 0.0001, Table 2). Importantly, no statistical differences were observed in C_min_, which means values were within the therapeutic range in all the three groups (<3 µg/mL). When analyzing the conventional regimen, statistical differences were identified only between the oncology patients under chemotherapy and the non-oncologic control group (*p* < 0.01, Table 2), with the former presenting lower C_max_ that may considerably compromise the therapeutic effect of amikacin.

Amikacin clearance was significantly higher in oncology patients under chemotherapy in relation to those without chemotherapy and from the control group (3.902, 3.098 and 2.778 mL/min, respectively, Table 2), suggesting that the drug is cleared faster in patients under chemotherapy. This is corroborated by its shorter half-life time (4.500 h) relative to the group of patients without chemotherapy (5.550 h) and with no cancer diseases (6.950 h). In opposition, no statistical differences were found among the distribution volume and elimination constant values of the three groups (*p* > 0.05, Table 2).

Since patients had received amikacin and chemotherapy within different interval times, it was herein tested, for the first time, whether this lag time could determine the pharmacokinetics of amikacin. In this regard, oncology patients were divided and classified according to the time between administration of amikacin and chemotherapy (Table 3). It stood out that amikacin clearance decreases as the lag time between the administration of amikacin and chemotherapy increases, becoming statistically significant when the time was longer than 30 days. The clearance found in the group with a lag time higher than 90 days (2.677 mL/min, Table 3) was similar to that observed in the control group (2.778 mL/min, Table 2). On the other hand, the highest half-life time of amikacin was observed in the same group. The administered volume of saline remained similar between all groups and no statical differences were found.

### 2.3. Amikacin Pharmacokinetics in Subpopulations

Considering the differences observed between oncology patients with and without chemotherapy and the control group regarding their mean age and renal function as well as the well-known impact of age and kidney function on the pharmacokinetics of aminoglycosides, the three groups were divided according to patient age (Section 2.3.1) and creatinine clearance (Section 2.3.2). Due to the small number of oncology patients with and without chemotherapy under conventional administration regimen (Figure 1), subpopulations were created enrolling only the patients under an extended interval regimen.

#### 2.3.1. Elderly

As demonstrated in the previous sections (Section 2.1 and Section 2.2, Table 1 and Table 2), the statistical differences between control patients and cancer patients regarding their age was notable; within the latter group, the differences between the age of oncology patients with and without chemotherapy was also relevant (Table 2). In fact, being elderly is closely linked to the loss of biological and physiological functions, which may compromise drugs’ pharmacokinetics. With this purpose, the two subpopulations of cancer patients and the control group were subdivided according to their age: 20–44, 45–64 and 65–85 years old. Their respective administered daily dose, amikacin concentrations and pharmacokinetics are summarized in Table 4 as well as the statistical differences between control patients, oncology patients with chemotherapy and without chemotherapy, within each age subpopulation.

According to Table 4, in the same age group, cancer patients were administered with higher doses than non-cancer patients (*p* < 0.0001). The unique exception were the patients without chemotherapy at the age range of 45–65 years old. Moreover, the doses administered to oncology patients with chemotherapy were higher than those administered without chemotherapy, corroborating the results reported in Section 2.2. It is interesting to emphasize that, in the elderly group (65–85 years old), no statistical differences were found between the doses administered to both oncology groups, suggesting that the impact of ageing is stronger than chemotherapy.

In spite of the increased doses administered to oncological patients under chemotherapy, C_min_ and C_max_ tended to be similar to or lower than those observed in oncology patients without chemotherapy. In fact, and also when analyzing dose-normalized concentrations, no statistical differences were detected between the two groups of oncology patients and the control group (Table 4), probably because of the increased mean values found for amikacin clearance in the group of patients with chemotherapy (Table 4). In this regard, independent of the age, the highest amikacin clearance was found in the chemotherapy group and the lowest value in the elderly, with statistical differences reported (Table 4). Complementarily, the mean half-life time of amikacin was lowest in patients with chemotherapy, followed by oncological patients without chemotherapy and non-oncological patients, particularly in the groups of 45–64 and 65–85 years of age, in which statistical differences were registered (Table 4). On the other hand, the youngest group exhibited very similar clearance and half-life times, with no statistical differences. 

Although it has not been specified in Table 4, since it is out of the scope of the present paper, statistical differences were identified between young adults, adults and the elderly. Indeed, the average daily doses of administered amikacin decreases with age (*p* < 0.001), regardless of whether the patient is oncological or not, or whether or not they are undergoing chemotherapy. Moreover, C_min_, which is a biomarker of amikacin accumulation and its toxicity, tended to increase with age. For instance, in oncology patients with chemotherapy, the values increased from 1.223 to 2.443 µg/mL, while in the control group it increased from 1.557 to 4.373 µg/mL (Table 4). Compared with the youngest group, the half-life time of amikacin was statistically superior in elderly oncology patients with chemotherapy (*p* < 0.001), without chemotherapy (*p* < 0.0001) and elderly non-oncology patients (*p* < 0.0001).

#### 2.3.2. Renal Impairment

Due to the high variability observed regarding amikacin elimination in cancer patients within each age group (Section 2.3.1), other factors are expected to compromise the pharmacokinetics of amikacin, including patient renal function since amikacin is almost exclusively eliminated by glomerular filtration. Therefore, serum creatinine was herein used to estimate glomerular filtration rate and renal function since it is entirely cleared by glomerular filtration. Based on the values of serum creatinine, creatinine clearance was estimated, applying the Cockroft and Gault equation, and patients were distinguished in 5 subpopulations regarding their renal function defined as follows, in accordance with [33,34]:

- ClCr < 30 mL/min/1.73 m^2^: Severe and terminal chronic renal impairment
- ClCr: 30–59 mL/min/1.73 m^2^: Moderate chronic renal impairment
- ClCr: 60–89 mL/min/1.73 m^2^: Mild chronic renal impairment
- ClCr: 90–120 mL/min /1.73 m^2^: Normal renal function
- ClCr ≥ 120 mL/min /1.73 m^2^: Renal lesion with normal renal function

The administered daily dose, concentrations and amikacin pharmacokinetics for these five subpopulations are summarized in Table 5 as well as the statistical differences between control patients, oncology patients with chemotherapy and oncology patients without chemotherapy. Accordingly, within each subpopulation, cancer patients were always administered with statistically significant higher daily doses. Importantly, dose differences increased as ClCr decreased. For instance, in patients with ClCr lower than 30 mg/mL/1.73 m^2^, the mean daily doses administered to oncology patients under chemotherapy and non-oncology patients were 1125 and 662.7 µg/mL, respectively, while, in patients with ClCr higher than 120 mL/min/1.73 m^2^, the mean daily doses were 1279 and 1067 µg/mL. Moreover, the dose was statistically lower in patients with compromised renal function (*p* < 0.001) in relation to those with normal function, but it was enhanced in patients with ClCr higher than 120 mL/min/1.73 m^2^ (*p* < 0.0001). However, even when administering lower doses, the C_max_ and C_min_ of amikacin were progressively higher when renal function decreases. Specifically, C_min_ was critically enhanced in patients with ClCr lower than 30 mL/min/1.73 m^2^, attaining values almost 10-fold of those observed in patients with normal function (12.05 and 1.318 µg/mL in oncology patients with chemotherapy, 14.16 and 1.1547 µg/mL in oncology patients without chemotherapy, 10.76 and 1.824 µg/mL in control patients, Table 5).

Identically to Section 2.3.1, within each subpopulation of ClCr, amikacin clearance and half-life time were statistically different and depend on whether the patient has an oncological disease or not and also if the patient is under chemotherapy or not. With exception of the subpopulation with ClCr lower than 30 mL/min/1.73 m^2^, the clearance was increased in all chemotherapy groups, presenting statistical differences in relation to the group without chemotherapy and the non-oncology patients. Nonetheless, the subpopulation with ClCr lower than 30 mL/min/1.73 m^2^ revealed the highest half-life time in patients under chemotherapy (18.13 min). In opposition, the remaining subpopulations demonstrated that the half-life time tended to be lower in patients with chemotherapy, with statistical differences in relation to non-oncology patients only in the subpopulations of 60–89 mL/min/1.73 m^2^ (*p* < 0.0001, Table 5).

## 3. Discussion

Intravenous amikacin is the antibiotic of choice for the treatment of Gram-negative infections, namely, those in neutropenic oncology patients [14]. Due to its intrinsic hydrophilic characteristics, amikacin is distributed almost exclusively in the volume corresponding to extracellular fluids; it does not undergo metabolism and is, hence, eliminated in its unchanged form mainly through glomerular filtration. Its narrow therapeutic range may expose the patient to toxicity and/or ineffectiveness, demanding an accurate TDM protocol for all patients. In turn, the specific physiological changes, aggressive pharmacological therapies and immunosuppression state place oncology patients under an increased risk of developing bacterial infections and large intra- and inter-individual variability in drug exposure, hampering the prediction of their dose-response [35]. Patients with malignant tumors represent a critical population, and inadequate empirical antibacterial therapy increases infection-related morbidity and mortality. In addition, pharmacokinetic parameters exhibit different characteristics compared to non-cancer patients, making the optimization of drug dosing and TDM essential [36].

However, studies are lacking comparing pharmacokinetic parameters of amikacin, in adult patients with tumors relative to non-oncology adult patients in Europe and overseas. In fact, to the best of our knowledge, there is currently only one pharmacokinetic populational study regarding amikacin in oncology patients. It enrolled 28 patients with haematological malignancies and dates 1999 [37]. No studies enrolling neutropenic cancer patients are currently reported. Herein, we performed a large-scale retrospective study that specifically examined the effects of tumor diseases on the pharmacokinetics of amikacin and investigated factors that contribute to amikacin pharmacokinetics in neutropenic cancer patients.

Firstly, we demonstrated that tumoral disease, independently of its type, was a factor affecting the clearance and half-life time of amikacin based on the results obtained by comparing drug pharmacokinetic parameters in non-oncology patients with oncology patients (Table 1). The clearance of amikacin is significantly increased in cancer patients (3.709 mL/min) in comparison to the control group (2.778 mL/min). Both groups presented CL mean values within the range reported in other investigations enrolling non-neutropenic patients [16,38,39]. Consequently, drug half-life time shortened from 6.950 to 4.750 h. Our results suggest that the increased clearance of amikacin may result from the fact that oncology patients are under a more severe inflammatory state than non-oncology patients, as evidenced by their mean value of C-reactive protein (CRP, 14.59 and 7.770 mg/L, respectively, Table 1). This inflammation seems to lead to systemic inflammatory response syndrome, which is characterized by vasodilatation, capillary leakage, high cardiac output and increased blood flow to major tissue, including the kidneys [40]. As amikacin is mainly renally excreted, its increased clearance is highly probable to result from the enhanced renal blood flow. On the other hand, the multiple amine groups of amikacin confer a cationic charge at physiologic pH, promoting its binding to anion phospholipids within the proximal tubule cell membrane in a saturable, electrostatic manner [41,42]. Amikacin is a P-glycoprotein substrate and probably also substrate of renal organic anion/cation transporters [43] that are activated by inflammatory molecules such as TNF -α, which are usually increased in oncology patients [36]. Activation of renal tubule cell membrane transporters promotes drug extrusion and elimination, probably justifying the increased clearance herein observed for amikacin in relation to non-oncologic patients.

Few previous studies have also shown that malignant tumors themselves may increase the clearance of other antibiotics [44], with some authors suggesting that the dose should be enhanced in 50% of cancer patients [45]. In fact, we herein demonstrated that the administered dose of amikacin significantly enhanced in oncology patients in relation to the control group (1170 and 868.6 mg, respectively, Table 1). Nonetheless, the percentage of oncological patients that successfully achieved the therapeutic range target (given by C_max_) was very low, with only 8.540% achieving the target level higher than 60 mg/L. On the other hand, 85.05% of oncological patients were within the pre-defined therapeutic range (vs. 63.00% of the non-oncology patients), highlighting that the higher administered dose in oncology patients does not seem to compromise amikacin accumulation or treatment safety. Our research strongly suggests that cancer patients require a higher initial dose of amikacin, indicating the necessity to design availably guidelines for amikacin dose individualization in cancer patients. 

To clarify whether the concentrations and pharmacokinetics of amikacin were affected by chemotherapy and the lag time between both treatments, oncology population was divided into patients without and with chemotherapy subpopulations (Table 2). Among the three subpopulations, non-oncology patients revealed the lowest value of clearance and the highest half-life time for amikacin, corroborating the findings for oncology and non-oncology groups. Furthermore, the mean values of C_max_ remained below therapeutic ranges, while C_min_ seemed to be increased in oncology patients without chemotherapy (9.050 and 2.245 mg/mL in conventional and interval extended dosing regimens, respectively) compared to oncology patients under chemotherapy (3.525 and 1.746 mg/mL) and non-oncology patients (8.827 and 1.650 mg/mL). Table 2 highlights that chemotherapy influences the concentrations of amikacin and its pharmacokinetics. 

Therefore, we investigated, for the first time, the impact of the temporal period between amikacin and chemotherapy administrations in the concentrations and pharmacokinetics of amikacin (Table 3). Statistical differences were reported amongst the several groups, evidencing that the clearance of amikacin decreases as the time between both administrations enhances: 3.932 mL/min when drugs were simultaneously administered and 2.677 mL/min when the lag time was superior to 90 days. In opposition, C_max_ and C_min_ increased as well as the half-life time. We therefore suggest that oncology patients that were under chemotherapy until 30 days should receive an increased dose of amikacin. In this context, we hypothesized whether this could result from a hyperdynamic circulation that is often observed in critical patients and increases the level of renal fluid, the glomerular filtration rate [46,47] and consequently the clearance of amikacin. However, the patients herein enrolled were not admitted in intensive care units and the volume of daily administered saline was not statistically different among the five subpopulations (Table 3), suggesting that additional factors can be affecting amikacin clearance. 

Bearing in mind that age is one of the most important factors influencing renal function [48] and that we found age and ClCr accounting for the wide range of the trough and maximum concentrations of amikacin (Table 2 and Table 3), subpopulations were created regarding patient age (Table 4) and renal function (Table 5). Hence, the impact of age in the concentrations and pharmacokinetics of amikacin was herein confirmed. Moreover, within each age subpopulation, the comparative studies corroborated that clearance of amikacin was higher in oncologic patients, particularly under chemotherapy. This analysis “neutralized” the influence of age and led to the conclusion that, not only oncological pathology, but also chemotherapy promotes an increase in the administered doses of amikacin. Similarly, within each subpopulation created in accordance with patient renal function, oncologic patients under chemotherapy are administered with higher doses than those without chemotherapy and no oncology pathologies. However, all of them revealed C_max_ and C_min_ progressively higher with the decrease of renal function, suggesting a greater predisposition of patients with ClCr lower than 60 mL/min to accumulate amikacin and develop toxic effects. Renal function clearly determined the amikacin clearance and concentrations, reducing the differences between oncology and non-oncology patients and between oncology patients with and without chemotherapy.

## 4. Materials and Methods

### 4.1. Study Design, Patients and Data Collectoin

The present retrospective study enrolled the clinical dataset from adult neutropenic patients admitted in Centro Hospitalar e Universitário de Coimbra (CHUC, EPE, Coimbra, Portugal) who had received at least one amikacin administration from February 2008 to December 2016 and underwent amikacin TDM, exhibiting, at least, one peak and trough concentration measurement. Exclusion criteria were age younger than 18 years, burns or cystic fibrosis (because of increased distribution volume) and admission in intensive care units. The study was approved by the Ethics Committee of Faculty of Medicine of University of Coimbra that waived patient consent as it was a noninterventional study based on data from routine patient care that was then retrospectively collected.

Two groups were initially created: the test group composed of 354 neutropenic oncology patients and the control group with 275 neutropenic non-oncology patients. The test group was divided in two subgroups regarding whether chemotherapy had been administered up to 3 months before amikacin administration (group named “with chemotherapy) or not (group named “without chemotherapy”).

Demographic and biological data, including age, sex, height, and total body weight (TBW), complete blood count, urea, creatinine, bilirubin, total protein and albumin concentrations were recorded. BMI and ideal body weight (IBW) were calculated [49,50]. ClCr was estimated with resort to the Cockcroft and Gault equation by using TBW [51]. Volume of administered saline was also compiled.

### 4.2. Sampling Procedure and Analytic Method for Amikacin Quantification

The amikacin initial dose was administered intravenously as a 30-min infusion with an electric syringe. Peak and trough samples are routine daily collected in steady state, at 1 h after the infusion stopped and before the next planned amikacin administration, respectively. The exact administration and sampling times were recorded for all patients. Amikacin concentrations were determined in the Laboratory of Clinical Pathology of CHUC, EPE, resorting to a validated fluorescence polarization immunoassay with the TDx analyzer (Abbott Laboratories, Abbott Park, IL, USA). The calibration range was between 0 and 50 mg/L. Samples with concentrations higher than 50 mg/L were diluted with Dillution buffer following the Dilution Protocol described by the manufacturer.

### 4.3. Pharmacokinetic Analysis and Endpoints

Plasma amikacin concentrations were analyzed by using PKS package, employing a non-linear regression method and one-compartmental open models with first-order elimination. The following pharmacokinetic variables were calculated for each patient: apparent volume of distribution, half-life time, elimination constant and clearance.

Amikacin levels measured 1 h (=peak) after perfusion and immediately before the next administration (trough) were considered the target concentrations.

### 4.4. Statistical Analysis

Statistical analyses were performed using the IBM SPSS 24.0 for the Windows NT software package. Descriptive statistics were computed for study variables. A Kolmogorov–Smirnov test was used, and histograms and normal-quantile plots were examined to demonstrate the normality of distribution of continuous variables. Demographics and clinical differences between oncology and non-oncology (sub)populations were assessed by using Student’s T test or ANOVA followed of Tukey’s test, as appropriate. A value of *p* < 0.05 was considered to be statistically significant.

## 5. Conclusions

It was herein demonstrated for the first time that tumor diseases increase clearance of amikacin and reduce its half-life time, allowing higher doses to be administered to neutropenic oncology patients. Nevertheless, even when increasing the dose, most oncology patients were at sub-therapeutic levels, but C_min_ was far away from being toxic, proving that higher amikacin doses can be administered to malignancy patients. Moreover, amikacin dose enhancement should be higher if the oncology patient is undergoing chemotherapy. Toxicity risk is expected to be reduced as C_min_ are not as high as those observed in cancer patients without chemotherapy.

The data analysis herein reported is clinically useful for predicting and estimating the appropriate dose of amikacin in oncologic populations, and it can be used to develop pharmacokinetic models that allow amikacin therapeutic individualization. It also remained clear that TDM should be performed at an early stage even though there is an increased risk of underestimating the concentrations, since early TDM implementation may not guarantee that the steady state has been attained.

## Figures and Tables

**Table 1:** Characteristics of patients and therapeutic drug monitoring included in the analysis. Values are expressed as mean ± standard deviation (minimum–maximum values) unless reported otherwise.

	Control Group	Test Group
**Sex** (female/male) ^a^	36.00/64.00 % (99/176)	43.50/56.50 % (154/200)
**Age** (years)	70.90 ± 14.20 (25–85)	54.00 ± 15.10 (20–85) ****
Frequency Distribution ^a^		
20–44 years old	9.090% (*n* = 25)	29.94% (*n* = 106)
45–64 years old	13.82% (*n* = 38)	43.50% (*n* = 154)
65–85 years old	77.09% (*n* = 212)	26.56% (*n* = 94)
**TBW** (kg)	67.35 ± 11.90 (40–120)	71.10 ± 11.77 (43–108) ***
**Height** (cm)	166.4 ± 8.310 (120–200)	166.8 ± 8.362 (148–189)
**IBW** (kg)	58.60 ± 7.87 (16.16–88.60)	58.23 ± 7.730 (41.52–78.64)
**BMI** (kg/m^2^)	24.31 ± 3.860 (14.59–42.97)	25.58 ± 4.180 (17.01–44.36) ***
Frequency Distribution ^a^		
<18.50	5.090%	1.900%
18.5–24.99	61.09%	52.94%
25–29.99	27.27%	33.04%
30–34.99	4.730%	8.480%
35–39.99	1.090%	3.110%
≥40.00	0.730%	0.520%
**ClCr** (mL/min)	79.15 ± 46.89 (1.95–397.6)	89.32 ± 34.04 (4.77–235.31) ****
**CRP** (10 mg/L)	7.770 ± 9.420 (0.060–138.3)	14.59 ± 11.35 (0.140–70.15) ****
**Daily Dose** (mg)	868.6 ± 383.5 (250.0–1750)	1170 ± 294.1 (500–2000) ****
**Treatment duration** (days)	13.70 ± 11.40 (3.000–141.0)	11.30 ± 0.8000 (2.000–46.00)
**Nº PK monitoring per patient**	2.200 ± 1.500 (1.000–14.00)	1.600 ± 0.9000 (1.000–6.000)
**Nº concentrations per patient**	4.400 ± 3.000 (2.000–28.00)	3.300 ± 1.800 (2.000–12.000)
**Saline volume daily administered** (L)	1.350 ± 1.080 (0.0000–5.100)	1.550 ± 0.6800 (0.0000–4.500) ****
**C_min_** (mg/L)		
Conventional regimen	8.827 ± 9.460 (0.0500–90.00)	4.216 ± 4.977 (0.0500–16.70)
Extended interval regimen	1.650 ± 0.9192 (0.0500–42.90)	1.867 ± 2.880 (0.0500–25.40)
**C_min_/dose**		
Conventional regimen (320)	0.0136 ± 0.8898 (0.0001–0.18)	0.0070 ± 1.2688 (0.0001–0.0334)
Extended interval regimen (280)	0.0047 ± 0.8891 (0.0000–0.0572)	0.0016 ± 1.2682(0.0000–0.0400)
**C_max_** (mg/L)		
Conventional regimen	29.07 ± 12.99 (9.900–107.0)	21.78 ± 6.639 (12.00–36.80)
Extended interval regimen	37.00 ± 17.07 (13.20–130.6)	44.00 ± 14.26 (13.00–95.40) ****
**C_max_/dose**		
Conventional regimen	0.0532 ± 0.8726 (0.0088–0.2092)	0.0367 ± 1.2592 (0.0231–0.0666) *
Extended interval regimen	0.0443 ± 0.8179 (0.0148–0.142)	0.0418 ± 1.2587 (0.0098–0.0914)
**Vd** (L/kg)	0.3764 ± 0.1775 (0.1007–1.645)	0.3755 ± 0.1261 (0.1876–1.731)
**CL** (L/h)	2.778 ± 1.476 (0.4213–9.838)	3.709 ± 1.639 (0.5017–15.26) ****
**Ke** (h^−1^)	0.1406 ± 0.0836 (0.0158–0.6463)	0.1744 ± 0.0589 (0.0089–0.5769) ****
**t_1/2_** (h)	6.950 ± 5.210 (1.100–43.80)	4.750 ± 4.080 (1.200–78.20) ****

^a^ Results are expressed as relative frequency (absolute frequency). BMI, body mass index; Cl, clearance; ClCr, creatinine clearance; CRP, C-reactive protein; IBW, ideal body weight; Ke, constant of elimination; PK, pharmacokinetic; t_1/2_, half-life time; TBW, total body weight; Vd, volume of distribution. * *p* < 0.05; *** *p* < 0.0002; **** *p* < 0.0001 through Student’s *t* test.

**Table 2:** Dose-normalized peak (C_max_) and trough (C_min_) concentrations and pharmacokinetic parameters observed in neutropenic oncology patients with and without chemotherapy compared to the control group. Values are expressed as mean ± standard deviation (minimum–maximum values) unless otherwise reported.

	Test Group		
	With Chemotherapy	Without Chemotherapy	Control Group
**Age** (years)	51.74 ± 15.28 (20–83)	56.78 ± 15.24 (23–85)	70.90 ± 14.20 (25–85)
**Daily dose** (mg)	1183 ± 298.4 (500.0–2000)	1130 ± 277.3 (500.0–1750)	868.6 ± 383.5 (250.0–1750)
**C_min_** (mg/L)			
Conventional regimen	3.525 ± 4.340 (0.0500–16.70) *	9.050 ± 8.556 (3.000–15.10)	8.827 ± 9.460 (0.0500–90.00)
Extended interval regimen	1.746 ± 2.842 (0.0500–25.40)	2.245 ± 2.976 (0.0500–20.10)	1.650 ± 0.9192 (0.0500–42.90)
**C_min_/Dose** Conventional regimen Extended interval regimen	0.0017 ± 0.0031 (0.0000–0.0334)	0.0024 ± 0.0042 (0.0000–0.0400)	0.0094 ± 0.0146 (0.0000–0.1800)
**C_max_** (mg/L) Conventional regimen Extended interval regimen			
	21.43 ± 5.971 (12.00–33.30) **	24.20 ± 13.58 (14.60–33.80)	29.07 ± 12.99 (9.900–107.0)
	43.00 ± 13.63 (15.10–82.20) ****	47.07 ± 15.71 (13.00–95.40) ****	37.00 ± 17.07 (13.20–130.6) ****
**C_max_/Dose** Conventional regimen Extended interval regimen	0.0365 ± 0.0109 (0.0100–0.0900)	0.0422 ± 0.0013 (0.01–0.090)	0.0491 ± 0.0203 (0.010–0.210)
**Vd** (L/kg)	0.3783 ± 0.1104 (0.1921–0.8480)	0.3668 ± 0.1667 (0.1876–1.7314)	0.3764 ± 0.1775 (0.1007–1.645)
**CL** (L/h)	3.902 ± 1.705 (0.5017–15.26) ****	3.098 ± 1.233 (0.7300–9.340)	2.778 ± 1.476 (0.4213–9.838)
**Ke** (h^−1^)	0.1800 ± 0.0603 (0.017–0.58)	0.1570 ± 0.0500 (0.0089–0.3029)	0.1406 ± 0.0836 (0.0158–0.6463)
**t_1/2_** (h)	4.500 ± 2.790 (1.200–39.90) ****	5.550 ± 6.620 (2.300–78.20) ****	6.950 ± 5.210 (1.100–43.804) ****

CL, clearance; Ke, constant of elimination; t_1/2_, half-life time; Vd, volume of distribution. * *p* < 0.05 in relation to control group; ** *p* < 0.01 in relation to control group; **** *p* < 0.0001 in relation to the other two groups through two-way ANOVA analysis followed by Tukey’s multiple comparison test.

**Table 3:** Dose-normalized peak (C_max_) and trough (C_min_) concentrations and pharmacokinetic parameters observed in neutropenic oncology patients with registration of chemotherapy. The time interval between amikacin therapy and chemotherapy is registered as ∆t. Only patients under extended interval regimen was enrolled. Values are expressed as mean ± standard deviation (minimum–maximum values).

	Simultaneous Amikacin and Chemotherapy	∆t < 15 Days	15 ≤ ∆t ≤ 30 Days	31 ≤ ∆t ≤ 90 Days	∆t > 90 Days
**Age** (years)	50.12 ± 17.72 (20–78)	50.91 ± 14.80 (20–83)	55.00 ± 14.30 (26–85)	55.70 ± 15.40 (23–79)	61.60 ± 9.200 (48–75)
**Daily dose** (mg)	1175 ± 308.4 (500.0–1750) *	1190 ± 301.0 (500.0–2000)	1158 ± 256.5 (500.0–1750)	1134 ± 268.7 (500.0–1750) **	1100 ± 341.8 (500.0–1750) **
**Saline daily volume** (L)	1.630 ± 0.8000 (0.0000–4.000)	1.520 ± 0.7000 (0.0000–4.500)	1.420 ± 0.5000 (0.5000–2.100)	1.550 ± 0.6000 (0.5000–3.000)	1.440 ± 0.6100 (0.5000–2.500)
**C_min_** (mg/L)	1.996 ± 4.055 (0.0500–25.40)	1.76 ± 2.85 (0.05–23.90)	1.51 ± 1.75 (0.05–9.30)	2.07 ± 2.24 (0.05–11.0)	3.51 ± 4.65 (0.05–20.00)
**C_min_/Dose**	0.0023 ± 0.0053 (0.0008–0.0100)	0.0017 ± 0.0029 (0.00003–0.0239)	0.0013 ± 0.0016 (0.00005–0.00853)	0.0019 ± 0.002 (0.00004–0.009)	0.0042 ± 0.0078 (0.00004–0.040)
**C_max_** (mg/L)	41.51 ± 14.83 (17.20–75.00)	43.73 ± 13.62 (15.10–95.40)	44.67 ± 15.99 (18.80–88.20)	45.60 ± 14.61 (13.00–86.10)	50.50 ± 16.02 (22.30–83.70)
**C_max_/Dose**	0.0358 ± 0.0109 (0.0100–0.0700)	0.0369 ± 0.0116 (0.0100–0.0900)	0.0388 ± 0.0115 (0.0200–0.0700)	0.0413 ± 0.0131 (0.0100–0.0900)	0.0446 ± 0.0096 (0.0300–0.0700)
**Vd** (L/kg)	0.3821 ± 0.1008 (0.1943–0.7060)	0.3773 ± 0.1143 (0.1936–0.8480)	0.3705 ± 0.0888 (0.1921–0.5979)	0.3559 ± 0.1044 (0.2028–0.8251)	0.9704 ± 0.2870 (0.1876–1.731)
**CL** (L/h)	3.932 ± 2.289 (0.5017–15.26) *	3.910 ± 1.632 (0.9303–13.04) *	3.705 ± 1.433 (0.7322–9.011) *	3.239 ± 1.355 (1.072–7.071) *	2.677 ± 1.099 (0.7475–5.221)
**Ke** (h^−1^)	0.1737 ± 0.0690 (0.0174–0.3234)	0.1804 ± 0.0577 (0.0297–0.5769)	0.1805 ± 0.0540 (0.0444–0.3029)	0.1631 ± 0.0516 (0.0366–0.2980)	0.1498 ± 0.0696 (0.0089–0.3152)
**t_1/2_** (h)	5.260 ± 5.030 (2.100–39.90) ****	4.400 ± 2.100 (1.200–23.30) ****	4.300 ± 1.900 (2.300–15.60) ****	4.840 ± 2.320 (2.300–19.00) ****	8.170 ± 14.080 (2.200–78.20)

CL, clearance; Ke, constant of elimination; t_1/2_, half-life time; Vd, volume of distribution. * *p* < 0.05 in relation to the group with ∆t > 90 days; ** *p* < 0.01 in relation to the group with ∆t < 15 days; **** *p* < 0.0001 in relation to the group ∆t > 90 days through two-way ANOVA analysis followed by Tukey’s multiple comparison test.

**Table 4:** Daily dose, dose-normalized peak (C_max_) and trough (C_min_) concentrations and pharmacokinetic parameters observed in neutropenic patients under extended interval regimen and classified in accordance with their age. Values are expressed as mean ± standard deviation (minimum–maximum values). Statistical comparisons are made between the three subpopulations defined by patient age.

		20–44 Years Old	45–64 Years Old	65–85 Years Old
**Daily dose** (mg)	With chemotherapy	1247 ± 311.9 (500.0–2000) ^a,b^	1181 ± 297.7 (500.0–2000) ^a,b^	1106 ± 263.0 ^a^
	Without chemotherapy	1177 ± 233.9 (1000–1750) ^a^	1134 ± 284.9 (500.0–1750)	1093 ± 291.5 (500.0–1500) ^a^
	Control group	783.3 ± 368.4 (250.0–1500)	1088 ± 418.5 (250.0–1750)	814 ± 350.9 (250.0–1750)
**C_min_** (mg/L)	With chemotherapy	1.223 ± 1.613 (0.0500–12.00)	1.741 ± 2.833 (0.0500–21.20)	2.443 ± 3.832 (0.0500–25.40)
	Without chemotherapy	1.098 ± 0.957 (0.0500–5.70)	2.106 ± 2.749 (0.0500–20.00)	3.180 ± 3.796 (0.0500–20.10)
	Control group	1.557 ± 1.244 (0.1000–5.40)	2.152 ± 2.438 (0.0500–13.30)	4.373 ± 5.453 (0.0500–42.90)
**C_min_/Dose**	With chemotherapy	0.0013 ± 0.0030 (0.0000–0.0334)	0.0015 ± 0.0024 (0.0000–0.0140)	0.0025–0.004 (0.0000–0.0250)
	Without chemotherapy	0.0010 ± 0.0009 (0.0000–0.0057)	0.0025 ± 0.0051 (0.0000–0.0400)	0.0031 ± 0.0039 (0.0000–0.0200)
	Control group	0.0052 ± 0.0071 (0.0001–0.0364)	0.0040 ± 0.0078 (0.0000–0.0702)	0.0114± 0.0161 (0.0000–0.1800)
**C_max_** (mg/L)	With chemotherapy	43.01 ± 13.79 (17.20–88.20)	42.82 ± 14.44 (15.10–83.90)	43.30 ± 12.06 (19.90–83.90)
	Without chemotherapy	48.15 ± 14.48 (2.180–95.40)	46.48 ± 16.30 (13.00–86.10)	47.25 ± 15.87 (17.50–84.00)
	Control group	43.26 ± 13.11 (23.00–64.00)	48.75 ± 19.38 (13.60–130.60)	45.03 ± 16.33 (13.20–99.30)
**C_max_/Dose**	With chemotherapy	0.0349 ± 0.0104 (0.0100–0.0700)	0.0362 ± 0.0112 (0.0100–0.0700)	0.0390 ± 0.0104 (0.020–0.0900)
	Without chemotherapy	0.0411 ± 0.0099 (0.0200–0.0700)	0.0413 ± 0.0143 (0.0100–0.0900)	0.0441 ± 0.0126 (0.020–0.0800)
	Control group	0.0438 ± 0.0139 (0.0200–0.0800)	0.0434 ± 0.0172 (0.0100–0.1300)	0.0513 ± 0.0213 (0.010–0.2100)
**Vd** (L/kg)	With chemotherapy	0.3744 ± 0.1189 (0.1940–0.8410)	0.3847 ± 0.1136 (0.1921–0.8480)	0.3727 ± 0.0924 (0.2000–0.7271)
	Without chemotherapy	0.3564 ± 0.1671 (0.2075–1.084)	0.3672 ± 0.1970 (0.1876–1.731)	0.3729 ± 0.1092 (0.1984–0.7401)
	Control group	0.3230 ± 0.1280 (0.1350–0.6120)	0.3466 ± 0.1182 (0.1007–0.7065)	0.3905 ± 0.1936 (0.1028–1.6450)
**CL** (L/h)	With chemotherapy	4.130 ± 1.679 (1.346–15.264) ^c^	4.087 ± 1.837 (0.5017–13.04) ^d^	3.3000 ± 1.3428 (1.0159–9.256) ^d^
	Without chemotherapy	3.414 ± 0.9206 (2.115–6.555)	3.2198 ± 1.216 (0.7474–5.733)	2.7128 ± 1.3553 (0.7322–9.341)
	Control group	3.871 ± 1.671 (0.9443–8.315)	3.0711 ± 1.563 (0.4213–9.571)	2.5790 ± 1.3639 (0.4271–9.838)
**Ke** (h^−1^)	With chemotherapy	0.1876 ± 0.0500 (0.0559–0.3234)	0.1872 ± 0.0664 (0.0174–0.5769)	0.1581 ± 0.0571 (0.0297–0.3018)
	Without chemotherapy	0.1856 ± 0.0035 (0.0624–0.2961)	0.1587 ± 0.0474 (0.0089–0.3029)	0.1363 ± 0.0558 (0.0444–0.2780)
	Control group	0.2355 ± 0.1547 (0.0633–0.6463)	1.160 ± 0.0899 (0.0161–0.5845)	0.1250 ± 0.0606 (0.0158–0.4088)
**t_1/2_** (h)	With chemotherapy	4.030 ± 1.490 (2.100–12.40)	4.400 ± 3.240 (1.200–39.90) ^a,b^	5.280 ± 3.130 (2.300–23.30) ^a,e^
	Without chemotherapy	3.970 ± 1.470 (2.300–11.10)	5.850 ± 9.160 (2.300–78.20)	6.110 ± 2.920 (2.500–15.60) ^a^
	Control group	4.080 ± 2.220 (1.100–11.00)	6.310 ± 5.430 (1.200–43.00)	7.430 ± 5.260 (1.700–43.80)

CL, clearance; Ke, constant of elimination; t_1/2_, half-life time; Vd, volume of distribution. ^a^ *p* < 0.0001 in relation to the control group; ^b^ *p* < 0.001 in relation to the group without chemotherapy; ^c^ *p* < 0.0001 in relation to the group without chemotherapy; ^d^ *p* < 0.05 in relation to the control group; ^e^ *p* < 0.05 in relation to the group without chemotherapy through two-way ANOVA analysis followed by Tukey’s multiple comparison test.

**Table 5:** Dose, dose-normalized peak (C_max_) and trough (C_min_) concentrations and pharmacokinetic parameters observed in neutropenic patients under extended interval regimen, classified in accordance with their clearance of creatinine (ClCr). Values are expressed as mean ± standard deviation (minimum–maximum values). Statistical comparisons are made between the five subpopulations defined by patient ClCr.

ClCr (mL/min/1.73 m^2^)		<30	30–59	60–89	90–120	>120
**Daily dose** (mg)	With chemotherapy	1125 ± 250.0 ^a,b^ (1000–1500)	1096 ± 301.4 ^a,b^ (500.0–1500)	1156 ± 269.0 ^b^ (500.0–1750)	1199 ± 303.5 ^b,e^ (500.0–1750)	1279 ± 311.4 ^b,e^ (500.0–2000)
	Without chemotherapy	937.5 ± 125.0 ^b^ (500.0–1000)	1071 ± 258.1 ^b^ (500.0–1500)	1166 ± 297.4 ^b^ (500.0–1750)	1131 ± 281.2 ^b^ (500.0–1750)	1208 ± 234.4 ^b^ (1000–1500)
	Control group	662.7 ± 283.7 (250.0–1500)	812.1 ± 333.5 (250.0–1750)	882.9 ± 360.8 (250.0–1750)	888.4 ± 371.3 (350.0–1750)	1067 ± 484.5 (250.0–1750)
**C_min_** (mg/L)	With chemotherapy	12.05 ± 12.13 (1.000–23.900)	3.390 ± 4.411 (0.0500–25.40)	1.375 ± 1.548 (0.0500–11.500)	1.318 ± 1.775 (0.0500–11.500)	1.122 ± 1.517 (0.0500–12.000)
	Without chemotherapy	14.16 ± 11.39 (0.0500–37.10)	2.707 ± 1.915 (0.0500–7.20)	2.279 ± 3.084 (0.0500–20.00)	1.155 ± 0.9891 (0.0500–5.700)	0.9667 ± 0.4207 (0.5000–2.10)
	Control group	10.76 ± 9.624 (1.000–42.90)	4.236 ± 4.166 (0.0500–21.60)	2.687 ± 2.041 (0.0500–10.80)	1.824 ± 1.312 (0.0500–6.20)	1.587 ± 1.959 (0.1000–13.20)
**C_min_/Dose**	With chemotherapy	0.1021 ± 0.0108 (0.0010–0.0239)	0.0034 ± 0.0045 (0.0000–0.0254)	0.0014 ± 0.0030 (0.0000–0.0334)	0.00128 ± 0.018 (0.0000–0.0100)	0.0008 ± 0.0009 (0.0000–0.0060)
	Without chemotherapy	0.0107 ± 0.0085 (0.0000–0.0201)	0.0026 ± 0.0019 (0.0001–0.0072)	0.0025 ± 0.0056 (0.0000–0.0400)	0.0016 ± 0.0023 (0.0000–0.012)	0.0008 ± 0.0003 (0.0000–0.0017)
	Control group	0.0276 ± 0.0301 (0.0010–0.1800)	0.0109 ± 0.0124 (0.0000–0.0624)	0.0071 ± 0.0081 (0.0000–0.0428)	0.0053 ± 0.0068 (0.0000–0.0386)	0.0036 ± 0.0058 (0.0001–0.0364)
**C_max_** (mg/L)	With chemotherapy	47.58 ± 16.76 (29.40–70.00)	47.63 ± 13.29 (18.80–83.90)	42.89 ± 13.46 (21.40–88.20)	42.45 ± 13.29 (19.30–80.80)	40.12 ± 13.76 (15.10–75.00)
	Without chemotherapy	55.83 ± 28.71 (13.00–92.10)	52.47 ± 18.66 (17.50–95.40)	46.99 ± 14.83 (19.70–84.00)	42.20 ± 11.53 (21.80–68.70)	44.83 ± 12.28 (24.40–64.30)
	Control group	44.76 ± 20.43 (15.30–86.40)	46.88 ± 17.10 (13.20–99.60)	48.08 ± 19.15 (18.40–130.6)	43.84 ± 13.97 (16.10–75.30)	43.49 ± 13.75 (13.60–71.00)
**C_max_/Dose**	With chemotherapy	0.0450 ± 0.0100 (0.0300–0.0500)	0.0436 ± 0.0107 (0.0200–0.0900)	0.0368 ± 0.0104 (0.0200–0.0700)	0.0351 ± 0.0098 (0.0200–0.0700)	0.03168 ± 0.0099 (0.0100–0.0600)
	Without chemotherapy	0.0475 ± 0.0299 (0.0100–0.0800)	0.0492 ± 0.0142 (0.0300–0.0900)	0.0409 ± 0.0108 (0.0200–0.0600)	0.0377 ± 0.0102 (0.0200–0.0600)	0.0375 ± 0.0087 (0.0200–0.0500)
	Control group	0.0694 ± 0.0304 (0.0200–0.2100)	0.0534 ± 0.0163 (0.0100–0.1200)	0.0485 ± 0.0199 (0.0100–0.1400)	0.0421 ± 0.0123 (0.0200–0.0900)	0.0371 ± 0.0132 (0.0100–0.0800)
**Vd** (L/kg)	With chemotherapy	0.5502 ± 0.1911 (0.2993–0.7271)	0.3624 ± 0.0839 (0.2003–0.5764)	0.3768 ± 0.1106 (0.1921–0.8480)	0.3684 ± 0.0935 (0.1936–0.6629)	0.3983 ± 0.1351 (0.1943–0.8411)
	Without chemotherapy	0.4619 ± 0.1920 (0.3034–0.7401)	0.3187 ± 0.0775 (0.1948–0.5266)	0.3840 ± 0.2127 (0.1876–1.7314)	0.3840 ± 0.1618 (0.1936–1.0836)	0.3714 ± 0.1197 (0.2199–0.6449)
	Control group	0.4551 ± 0.2588 (0.1350–1.5039)	0.3688 ± 0.1732 (0.1497–1.6178)	0.3608 ± 0.1651 (0.1007–1.6450)	0.3716 ± 0.1702 (0.1354–1.6436)	0.3699 ± 0.1307 (0.1197–0.6943)
**CL** (L/h)	With chemotherapy	2.163 ± 1.684 (0.5017–4.329)	2.800 ± 1.321 ^c^ (1.016–9.256)	3.804 ± 1.232 ^d^ (1.346–7.309)	4.116 ± 1.578 ^b,c^ (1.056–9.012)	4.726 ± 2.177 ^a,b^ (2.189–15.264)
	Without chemotherapy	2.012 ± 1.864 (0.732–4.747)	2.453 ± 0.9877 (0.747–5.197)	3.065 ± 1.287 (0.943–9.341)	3.737 ± 1.006 (2.197–6.556)	3.735 ± 0.808 ^b^ (2.842–5.129)
	Control group	1.362 ± 0.7582 (0.4213–6.119)	2.121 ± 0.9682 (0.6379–6.119)	2.923 ± 1.396 (0.6780–9.341)	3.436 ± 1.518 (1.36–9.838)	3.871 ± 1.391 (1.495–8.051)
**Ke** (h^−1^)	With chemotherapy	0.0864 ± 0.0730 (0.0174–0.1573)	0.1454 ± 0.0619 (0.0366–0.2837)	0.1819 ± 0.0516 (0.0701–0.3818)	0.1894 ± 0.0522 (0.0448–0.3234)	0.1962 ± 0.0641 (0.0559–0.5769)
	Without chemotherapy	0.1038 ± 0.10355 (0.0444–0.2586)	0.1304 ± 0.0487 (0.0492–0.2775)	0.1543 ± 0.0526 (0.0089–0.3029)	0.1762 ± 0.0392 (0.0624–0.2961)	0.1824 ± 0.0230 (0.1310–0.2176)
	Control group	0.0709 ± 0.0676 (0.0158–0.5249)	0.1106 ± 0.0546 (0.0283–0.3086)	0.1483 ± 0.0614 (0.0433–0.4088)	0.1755 ± 0.1015 (0.0248–0.6463)	0.1854 ± 0.0958 (0.0401–0.6091)
**t_1/2_** (h)	With chemotherapy	18.13 ± 16.97 ^c^ (4.400–39.90)	5.950 ± 3.230 ^b^ (2.40–19.00)	4.140 ± 1.350 ^a,b^ (1.800–9.900)	4.080 ± 1.76 ^b^ (2.100–15.50)	3.860 ± 1.330 ^b^ (1.200–12.40)
	Without chemotherapy	10.87 ± 5.820 (2.700–15.60)	5.640 ± 2.360 ^b^ (2.50–14.10)	6.320 ± 10.23 ^d^ (2.300–78.20)	4.23 ± 1.54 ^b^ (2.30–11.10)	3.870 ± 0.580 ^b^ (3.200–5.300)
	Control group	14.54 ± 9.250 (1.300–43.80)	8.120 ± 4.570 (2.200–24.50)	5.540 ± 2.490 (1.700–15.70)	4.970 ± 2.850 (1.100–27.90)	4.590 ± 2.310 (1.100–17.30)

CL, clearance; ClCr, clearance of cretinine; Ke, constant of elimination; t_1/2_, half-life time; Vd, volume of distribution. ^a^ *p* < 0.0001 in relation to the group without chemotherapy; ^b^ *p* < 0.0001 in relation to the control group; ^c^ *p* < 0.05 in relation to the group without chemotherapy; ^d^ *p* < 0.05 in relation to the control group; ^e^ *p* < 0.001 in relation to the group without chemotherapy; through two-way ANOVA analysis followed by Tukey’s multiple comparison test.

## Data Availability

Data are unavailable due to privacy or ethical restrictions.
